# Construction and application of a smart hospital platform-based VTE risk management system

**DOI:** 10.3389/fpubh.2026.1876826

**Published:** 2026-07-13

**Authors:** Chengye Xu, Jiapei Yao, Liuyue Wang, Dongmei Xu, Yuqing Jiang, Xiaofei Wang

**Affiliations:** 1 School of Medicine, Hunan Normal University, Changsha, China; 2 School of Nursing, Hunan Normal University, Changsha, China; 3 Kiang Wu Nursing College of Macau, Macau, Macao SAR, China; 4 Department of Orthopedics, The Second People's Hospital of Changzhou, The Third Affiliated Hospital of Nanjing Medical University, Changzhou, China; 5 School of Nursing, Changzhou University, Changzhou, China

**Keywords:** clinical decision support, hospital information system, physician-nurse collaboration, risk assessment, risk management system, smart hospital platform, venous thromboembolism

## Abstract

**Objective:**

Venous thromboembolism (VTE) ranks as the third most prevalent cardiovascular disease after acute myocardial infarction and stroke, posing a significant risk to patient safety. Clinical challenges such as inadequate VTE awareness among healthcare professionals, low prevention rates, untimely and inaccurate risk assessments, improper grading of preventive measures, and insufficient physician-nurse communication necessitate the development of a more effective management approach. This study aimed to design and implement a smart hospital platform-based VTE risk management system and evaluate its impact on VTE and bleeding risk assessment times, thereby enhancing clinical VTE prevention and control.

**Methods:**

A prospective interventional study was conducted to compare outcomes before and after the implementation of the VTE risk management system. The study population comprised inpatients at a tertiary grade A hospital. The pre-implementation cohort included patients admitted from March 1 to June 30, 2024 (*n* = 5,763), while the post-implementation cohort consisted of patients admitted from March 1 to June 30, 2025 (*n* = 5,842). The VTE risk management system was structured into five functional modules: structured assessment, stratified decision-making, intelligent alerts, physician-nurse communication, and discharge follow-up. Evaluation metrics encompassed risk assessment efficiency, process quality indicators, clinical outcome measures, and VTE knowledge levels among healthcare professionals.

**Results:**

Following system implementation, the time required for nurses to complete VTE risk assessments was reduced from 101.90 ± 15.694 s to 37.18 ± 5.701 s (*p* < 0.001), while the time for physicians to complete bleeding risk assessments decreased from 138.08 ± 17.348 s to 50.00 ± 9.013 s (*p* < 0.001). The timeliness of VTE risk assessments improved from 71.0 to 92.0% (*p* < 0.001), bleeding risk assessment timeliness increased from 74.0 to 94.0% (*p* < 0.001), dynamic risk assessment rates rose from 78.0 to 95.0% (*p* < 0.001), and the accuracy of risk assessments improved from 64.0 to 92.0% (*p* < 0.001). Additionally, the correct implementation of intervention measures increased from 75.0 to 93.0% (*p* = 0.001). Promising trends were also observed in clinical outcomes: the incidence of VTE among inpatients decreased from 0.316‰ to 0.098‰ (*p* = 0.012), and bleeding incidence from 0.432‰ to 0.180‰ (*p* = 0.008). The average length of hospital stay was reduced from 9.7 ± 3.1 days to 8.3 ± 2.4 days (*p* < 0.001). Furthermore, VTE-related knowledge among healthcare professionals improved markedly, with awareness of VTE assessment scales increasing from 76.25 to 97.50% (*p* < 0.001), and the proportion of staff perceiving VTE prevention as a workload burden decreasing from 65.00 to 26.25% (*p* < 0.001).

**Conclusion:**

The VTE risk management system developed on a smart hospital platform demonstrated marked improvements in risk assessment efficiency and process quality indicators. These findings suggest that the system is feasible and can effectively support standardized VTE management in a real-world hospital setting. However, given the pre-post study design without a concurrent control group, the observed clinical benefits should be interpreted as preliminary trends rather than definitive evidence of effectiveness. Future randomized controlled trials are warranted to confirm the comparative efficacy of this integrated approach.

## 1 Introduction

Venous thromboembolism (VTE) is the third leading cardiovascular disease, following acute myocardial infarction and stroke. Its occurrence prolongs hospital stays, escalates healthcare costs, reduces patients’ quality of life, and poses a substantial threat to survival (1, 2). In China, the incidence of VTE among hospitalized patients is estimated at 17.5 per 100,000 (3), with 10% of at-risk patients also facing a high risk of bleeding (4). Moreover, a majority of hospitalized patients exhibit at least one VTE risk factor, including advanced age, prolonged immobility, active malignancy, prior VTE history, recent surgery or trauma, acute infection, heart or respiratory failure, obesity, and chronic medical conditions such as hypertension and diabetes (5). Timely VTE risk assessment and the implementation of preventive strategies can significantly reduce VTE incidence in hospitalized patients (6, 7). The adoption of information technology tools further enhances risk management efficiency for VTE prevention and treatment (8).

Despite these advancements, current clinical practice is hindered by inadequate VTE awareness among healthcare providers (9, 10), with low rates of appropriate preventive interventions (9.3% in surgical departments and 6.0% in medical departments) (11, 12). Additional barriers include untimely and inaccurate assessments, improper grading of preventive measures, poor communication between physicians and nurses, and insufficient follow-up management. In response, our hospital launched a VTE risk management system in July 2021. This smart hospital information platform, built upon the HIS, integrates a clinical decision support module and follows the design principles of “prioritized prevention, intelligent augmentation, and full closed-loop management,” aiming to integrate risk assessment, stratified intervention, and follow-up management into a standardized workflow. The system was implemented with the goal of enhancing clinical VTE prevention and control and effectively reducing both VTE and bleeding incidence.

## 2 Construction of the VTE risk management system based on a smart hospital platform

### 2.1 Establishment of a VTE prevention and management team

A multidisciplinary management team was established, comprising the hospital director, clinical department heads, and head nurses. The hospital director served as the team leader, overseeing VTE prevention and control initiatives, formulating relevant policies and workflows, and ensuring comprehensive quality control throughout the intervention process. A system development team was additionally formed, consisting of eight key members: one medical information specialist and one nursing information specialist, responsible for identifying system requirements, communicating functional needs, and designing risk management modules; one IT engineer, responsible for collecting system feedback, troubleshooting application issues, and providing operational training across different hospital departments; and five software development engineers, tasked with implementing and continuously optimizing system functionalities.

### 2.2 Design of VTE risk management system modules

#### 2.2.1 Structured assessment module

Healthcare professionals conduct risk assessments through the system’s computer or mobile interface, with real-time synchronization between both platforms. (1) VTE Risk Assessment: ① Assessment Tools: Nurses use the Caprini assessment scale for surgical patients and the Padua assessment scale for medical patients (10, 13). Although the Caprini scale can theoretically be applied to all hospitalized patients, it is primarily validated for surgical populations and contains numerous surgery-specific items; the Padua scale is specifically validated for medical inpatients and is recommended by international guidelines for this group. The combined use of both scales according to patient type ensures risk stratification accuracy and guideline conformity. The Caprini scale assigns weighted scores to nearly 40 risk factors, categorized as 1, 2, 3, or 5 points, with final classifications into four levels: very low risk (0 points), low risk (1–2 points), moderate risk (3–4 points), and high risk (≥ 5 points). The Padua scale assigns weighted values to 11 risk factors, categorized as 1, 2, or 3 points, with results classified into two levels: low risk (< 4 points) and high risk (≥ 4 points). After nurses select the relevant factors, the system calculates scores in real time and simultaneously updates risk levels in both physician and nurse interfaces. ② Assessment Timing: VTE risk assessments are performed within 24 h of admission, within 6 h postoperatively, before discharge, upon department transfer, and whenever risk factors change (10). (2) Bleeding Risk Assessment: Patients undergoing VTE risk assessment also undergo bleeding risk evaluation. ① Assessment Tool: A self-designed bleeding risk factor table, based on relevant guidelines and research (5, 14), includes 20 risk factors. The 20 bleeding risk factors are as follows [based on guidelines (5, 14)]:1. Active peptic ulcer.2. History of bleeding requiring hospitalization.3. Liver failure (INR > 1.5)4. Severe renal impairment (eGFR < 30 mL/min)5. Thrombocytopenia (< 50 × 10^9^/L)6. Uncontrolled hypertension (SB*P* > 160 mmHg)7. Recent major surgery or trauma (< 3 months)8. Concomitant antiplatelet therapy.9. Concomitant anticoagulant therapy.10. History of intracerebral hemorrhage.11. Intracranial tumor or vascular malformation.12. Active cancer with bleeding risk (e.g., bladder cancer, gastrointestinal tumor)13. Recent gastrointestinal bleeding (< 3 months)14. Advanced age (≥ 85 years)15. Anemia (Hb < 90 g/L)16. Liver cirrhosis with esophageal varices.17. von Willebrand disease or other coagulopathy.18. Recent extensive skin or mucosal bleeding (< 6 weeks)19. Acute pancreatitis.20. Uncorrected coagulopathy (PT or APTT > 1.5 times upper normal limit) Patients are classified as low risk (0 factors), moderate risk (1 factor), or high risk (≥2 factors). Once the attending physician selects relevant factors based on the patient’s condition, the system immediately updates the bleeding risk level in physician and nurse interfaces. ② Assessment Timing: Assessments are conducted when patients initiate or modify anticoagulant therapy, undergo surgery, become pregnant, or experience deteriorating conditions (10). (3) Assessment Result Organization: The system automatically records and organizes assessment data, including assessment time, scores, risk levels, and responsible personnel, providing a structured overview of the patient’s VTE-related risk profile. Nurses retrieve risk factors from electronic medical records, physician notes, laboratory results, and patient interviews. Factors not directly observable are auto-synced from physician entries to the nursing assessment form.

Surgical patients were defined as those who were scheduled for or underwent a surgical procedure during hospitalization including preoperative postoperative and interventional stages. Medical patients were those without surgical plans receiving primarily pharmacotherapy or conservative management. If a patient’s condition changed significantly due to infection heart failure immobilization or transfer to the ICU or if a medical patient later required surgery the system prompted a repeat VTE risk assessment. Patients undergoing invasive procedures such as central venous catheter placement endoscopic therapy or percutaneous biopsy were managed as surgical patients using the Caprini scale if the procedure was traumatic and required anesthesia or post-procedural bed rest.

#### 2.2.2 Stratified decision-making module

VTE prevention strategies were formulated based on expert consensus and relevant research (10, 15), and integrated into the stratified decision-making module. The system automatically recommends appropriate prevention measures based on patient risk assessment results: all patients receive basic prevention unless contraindicated; low-risk patients receive mechanical prophylaxis; moderate-risk patients receive pharmacological and/or mechanical prophylaxis; and high-risk patients are managed based on their bleeding risk level—those at low bleeding risk receive pharmacological prophylaxis, while those at moderate to high bleeding risk receive mechanical prophylaxis. The preventive strategies in this module were developed based on the ACCP-9 guidelines (14), the Chinese VTE Prevention and Treatment Guidelines, and multidisciplinary expert consensus, and were reviewed by our hospital’s VTE Prevention Committee. All recommendations are supported by evidence-based medicine including pharmacological prophylaxis for moderate-to-high risk patients with low bleeding risk as a Grade 1B recommendation. Once healthcare professionals select and save interventions, the system synchronizes these decisions with structured electronic medical records and medical orders.

#### 2.2.3 Intelligent alert module

The system provides high-risk identification alerts and assessment timing reminders based on patients’ VTE risk levels, diagnostic conditions, and disease progression. (1) High-Risk Identification Alerts: When a patient is classified as high risk for VTE, the system issues alerts in two ways across computer and mobile interfaces: ① A VTE high-risk log is displayed in the ward overview interface, compiling a list of patients marked with VTE high-risk identifiers. ② A red-bordered square identifier with the word “clot” appears below the patient’s bed card. These alerts are dynamically updated and shared between computer and mobile platforms. (2) Assessment Timing Reminders: The system sets predefined assessment timeframes according to VTE assessment protocols (10, 16). A yellow reminder box stating “Bed X requires VTE assessment” appears in the bottom right corner of smart physician and nursing terminals (mobile phones, computers) and disappears upon completion of the assessment.

#### 2.2.4 Physician-nurse communication module

A dedicated physician-nurse communication window is embedded within the smart medical platform’s physician and nursing interfaces on both computer and mobile devices. Clicking on the window opens a patient list on the left (sortable by bed number), a healthcare staff list on the right, and a central communication area supporting text, voice, images, photos,and video messages.enabling healthcare providers to share wound conditions, drainage characteristics, or imaging findings when discussing complex cases. Red dots on the window or patient names indicate new messages, while urgent messages trigger an automatic pop-up or patient name shake. Healthcare professionals can discuss complex VTE assessments and interventions, and can tag (@) VTE prevention experts for joint discussions in challenging cases. The window minimizes upon communication completion.

#### 2.2.5 Discharge follow-up module

Upon patient discharge, the follow-up module automatically retrieves patient information, including lab results, examination findings, and medical records from the smart hospital platform. Patients categorized as “VTE high risk” or “bleeding high risk” at discharge are automatically filtered for follow-up. Healthcare professionals conduct telephone follow-ups as needed, providing targeted guidance based on prior intervention measures and system-recorded patient histories. They address patient and family inquiries in layman’s terms and offer additional health education.

## 3 Clinical application of the VTE risk management system based on the smart hospital platform

### 3.1 Study subjects

A prospective interventional study was conducted to assess the system’s impact. The study population comprised inpatients at a tertiary grade A hospital in Changzhou City. The pre-implementation cohort consisted of patients admitted between March 1 and June 30, 2024 (*n* = 5,763), while the post-implementation cohort included those admitted between March 1 and June 30, 2025 (*n* = 5,842).

### 3.2 Application approach

The system was deployed across all inpatient departments in March 2025. Within 24 h of admission, nurses performed initial VTE risk assessments using computer or mobile terminals under physician supervision, while physicians completed bleeding risk assessments.”Based on assessment results, the system provided automated prevention recommendations, and healthcare professionals selected and implemented appropriate interventions. The system prompted reassessments at critical points, including post-surgery, changes in condition, and medication adjustments. Complex VTE prevention issues were addressed through the collaboration module. Before discharge, risk reassessments were conducted, and follow-up plans were formulated for high-risk patients.

To ensure smooth implementation, the research team conducted comprehensive training sessions for all hospital staff, including centralized and department-specific training. Illustrated operational guides and FAQs were provided. Each department designated 1–2 information specialists to oversee daily operations and report system-related issues. Weekly system usage reports were generated, and monthly VTE prevention and control quality analysis meetings were held.

### 3.3 Evaluation indicators

The following three core indicators were compared before (March 1 to June 30, 2024) and after (March 1 to June 30, 2025) the implementation of the VTE risk management system:

#### 3.3.1 Risk assessment efficiency indicators

Risk assessment time refers to the average time required for healthcare professionals to complete a VTE or bleeding risk assessment. One hundred patients were randomly selected before and after system implementation, with assessments jointly conducted by physicians and nurses.

#### 3.3.2 Process quality indicators

VTE risk assessment timeliness rate = (Number of patients completing VTE assessment within 24 h of admission/Total number of patients admitted during the same period) × 100%; Bleeding risk assessment timeliness rate = (Number of patients completing bleeding risk assessment within 24 h of admission/Total number of patients admitted during the same period) × 100%; Dynamic risk assessment rate = (Number of patients receiving risk assessments according to assessment timing / Total number of patients during the same period) × 100%; Risk assessment accuracy rate = (Number of patients whose risk assessment items match their actual conditions/Total number of patients during the same period) × 100%; Correct implementation rate of intervention measures = (Number of patients correctly implementing intervention measures / Total number of patients implementing intervention measures during the same period) × 100%. One hundred hospitalized patients were randomly selected before and after system implementation. Inspections and statistical analyses were conducted by two professional management group members.

#### 3.3.3 Clinical outcome indicators

VTE incidence = (Number of patients developing VTE during the statistical period / Total number of patients during the same period) × 1,000‰; Bleeding incidence = (Number of patients developing bleeding during the statistical period/Total number of patients during the same period) × 1,000‰; Length of hospital stay = Average number of days from patient admission to discharge.

#### 3.3.4 Healthcare professionals’ VTE knowledge level

Before and after system implementation, 80 physicians and nurses from our hospital were randomly selected and surveyed using a self-designed questionnaire to assess their VTE knowledge.

### 3.4 Statistical methods

Statistical analysis was performed using SPSS 26.0 software. After normality testing measurement data conforming to a normal distribution were expressed as mean ± standard deviation (x ± s) and compared using independent sample t-tests. Data not conforming to a normal distribution were expressed as median (interquartile range) [M(IQR)] and compared using Mann–Whitney U tests. Count data were expressed as frequency (*n*, %) and compared using chi-square (χ^2^) tests or Fisher’s exact probability method. Clinical outcome indicators were analyzed using Kaplan–Meier survival analysis, with confounding factors (such as age, gender, and underlying diseases) adjusted using Cox proportional hazards regression models. All statistical tests were two-sided, with *p* < 0.05 considered statistically significant. Data were verified and compiled from electronic records and system backend data by two professional management group members.

## 4 Results

### 4.1 Baseline characteristics

No statistically significant differences were observed between the two patient groups in terms of baseline characteristics, including age, gender, BMI, admission department distribution, comorbidities, and VTE risk factors (*p* > 0.05), indicating good comparability (Table 1).

**Table 1:** Comparison of baseline characteristics between the pre- and post-implementation groups.

Characteristic	Pre-implementation group (*n* = 5,763)	Post-implementation Group (*n* = 5,842)	Statistic	*p*-value
Age (years,^−^*x* ± *s*)	58.3 ± 15.7	59.1 ± 16.2	*t* = 1.462	0.144
Gender [*n* (%)]			*x*^2^ = 0.524	0.469
Male	3,116 (54.1)	3,129 (53.6)		
Female	2,647 (45.9)	2,713 (46.4)		
BMI (kg/m^2^, x̄±s)	24.3 ± 3.8	24.1 ± 3.6	*t* = 1.593	0.111
Admission department [*n* (%)]			*x*^2^ = 2.784	0.595
Internal medicine	2,534 (44.0)	2,621 (44.9)		
Surgery	2,051 (35.6)	2,022 (34.6)		
Obstetrics and gynecology	624 (10.8)	651 (11.1)		
Orthopedics	398 (6.9)	421 (7.2)		
Others	156 (2.7)	127 (2.2)		
Comorbidities [*n* (%)]				
Hypertension	1,917 (33.3)	1,982 (33.9)	*x*^2^ = 0.568	0.451
Diabetes	1,043 (18.1)	1,085 (18.6)	*x*^2^ = 0.452	0.501
Coronary heart disease	874 (15.2)	903 (15.5)	*x*^2^ = 0.209	0.648
Malignant tumors	635 (11.0)	672 (11.5)	*x*^2^ = 0.748	0.387
Stroke	412 (7.1)	434 (7.4)	*x*^2^ = 0.351	0.553
VTE risk factors [*n* (%)]				
Long-term bed rest	972 (16.9)	1,015 (17.4)	*x*^2^ = 0.537	0.464
Surgical history	1,623 (28.2)	1,594 (27.3)	*x*^2^ = 1.229	0.268
Obesity	807 (14.0)	836 (14.3)	*x*^2^ = 0.230	0.632
Fractures	263 (4.6)	284 (4.9)	*x*^2^ = 0.497	0.481
Oral contraceptives	118 (2.0)	132 (2.3)	*x*^2^ = 0.633	0.426

### 4.2 Comparison of risk assessment efficiency

Before system implementation, nurses required 101.90 ± 15.694 s to complete a VTE risk assessment, which decreased to 37.18 ± 5.701 s after implementation, showing a statistically significant difference (*t* = 24.516, *p* < 0.001). Similarly, physicians required 138.08 ± 17.348 s for a bleeding risk assessment before implementation, which decreased to 50.00 ± 9.013 s afterward, also showing a statistically significant difference (*t* = 28.494, *p* < 0.001).

### 4.3 Comparison of VTE process quality indicators

All VTE process quality indicators significantly improved following system implementation (*p* < 0.01) (Table 2).

**Table 2:** Comparison of VTE prevention and control process quality indicators before and after system implementation [*n* (%)].

Indicator	Pre-implementation group (*n* = 100)	Post-implementation group (*n* = 100)	*x* ^2^	*P*-value
VTE risk assessment timeliness rate	71 (71.0)	92 (92.0)	14.624	<0.001
Bleeding risk assessment timeliness rate	74 (74.0)	94 (94.0)	14.881	<0.001
Dynamic risk assessment rate	78 (78.0)	95 (95.0)	12.374	<0.001
Risk assessment accuracy rate	64 (64.0)	92 (92.0)	22.844	<0.001
Correct implementation rate of intervention measures	75 (75.0)	93 (93.0)	12.054	0.001

### 4.4 Comparison of clinical outcome indicators

After system implementation, the incidence of VTE among hospitalized patients decreased from 0.316‰ to 0.098‰ (*χ^2^* = 6.359, *p* = 0.012). The incidence of bleeding decreased from 0.432‰ to 0.180‰ (*χ^2^* = 7.000, *p* = 0.008). The average length of hospital stay was reduced from 9.7 ± 3.1 days to 8.3 ± 2.4 days, a reduction of 1.4 days (*t* = 12.476, *p* < 0.001).

### 4.5 Comparison of healthcare professionals’ VTE knowledge

Details of the comparison can be found in Table 3.

**Table 3:** Comparison of healthcare professionals’ VTE knowledge before and after system implementation (*n*, %).

Knowledge content	Pre-implementation group (*n* = 80)	Post-implementation group (*n* = 80)	*x* ^2^	*P*-value
Understanding of VTE scales	61 (76.25)	78 (97.50)	14.032*	<0.001
Necessity of assessment	56 (70.00)	68 (85.00)	5.161	0.023
Necessity of physician-nurse collaboration	53 (66.25)	75 (93.75)	18.906	<0.001
Helpfulness of information technology for prevention and treatment	46 (57.50)	71 (88.75)	21.432	<0.001
Belief that it increases clinical workload	52 (65.00)	21 (26.25)	24.210	<0.001

*Corrected chi-square test.

## 5 Discussion

While the improvements observed in this study were temporally associated with the system deployment, causal attribution should be made with caution. The pre-post design without a concurrent control group cannot fully account for secular trends in VTE awareness, concurrent hospital quality improvement initiatives, or the Hawthorne effect. Therefore, we frame our findings primarily as evidence of implementation feasibility and preliminary effectiveness, rather than definitive comparative efficacy.

### 5.1 System implementation improved VTE risk assessment efficiency

The results demonstrate that the VTE risk management system significantly reduced the time required for healthcare professionals to complete risk assessments, enhancing clinical efficiency and reducing workload. Similar to Hunt et al.’s findings (6), these results demonstrate that information technology tools can streamline assessments through structured processes and automated calculations. However, the efficiency improvements in this study were more pronounced, likely due to localized system design and a user-friendly interface. Neeman et al. (5) found that VTE risk assessment tools integrated with electronic health records (EHR) improved efficiency, though the extent of improvement was often constrained by system integration and hospital IT infrastructure. The smart hospital platform used in this study achieved higher integration and optimization, leading to greater efficiency gains. Additionally, Sannino et al. (17) reported that lightweight mobile applications enhance healthcare efficiency more effectively than traditional EHRs, aligning with this study’s dual-platform approach (computer and mobile). Future research should explore long-term adaptation to the system and its efficiency in different clinical scenarios.

### 5.2 System implementation improved VTE prevention and control process quality indicators

In this study, all VTE prevention and control process quality indicators improved significantly after system implementation, including VTE risk assessment timeliness rate, bleeding risk assessment timeliness rate, dynamic risk assessment rate, risk assessment accuracy rate, and correct implementation rate of intervention measures. These improvements reflect the system’s advantages in standardized assessment processes, intelligent reminders, and assisted decision-making. Compared to Kakkar et al. (4), our study achieved higher risk assessment and intervention implementation rates, possibly due to the integration of intelligent reminders and standardized workflows that reinforced adherence to guidelines. With respect to process quality indicators, studies outside China have yielded differing improvement patterns. Lau et al. (15) reported a 20% improvement in prevention implementation rates using a multi-module system in Canada, whereas this study demonstrated even greater gains. This may be attributed to different baseline levels, as some healthcare systems already have established VTE prevention protocols, leaving less room for improvement. Sutton et al. (18), in contrast, found that clinical decision support systems (CDSS) could achieve a correct intervention implementation rate of up to 95%, comparable to this study’s results. However, international studies often employ more complex risk stratification and real-time monitoring, requiring substantial IT investment. This study, in contrast, prioritized practical application and local resource optimization, achieving significant improvements with relatively lower resource input. Notably, while international research often separates VTE and bleeding risk assessments, this study integrated both within a single platform, providing a clinically relevant solution for balancing anticoagulation and bleeding risks.

### 5.3 System implementation improved clinical outcome indicators

Following system implementation, we observed reductions in the incidence of VTE and bleeding among inpatients, alongside a decreased average length of hospital stay. These changes suggest that the system may contribute to improved patient outcomes. However, given the low baseline event rates, with VTE at 0.316‰ and bleeding at 0.432‰, the observed absolute risk reductions were small, and the statistical significance may be sensitive to the small number of events. These findings should therefore be interpreted with particular prudence, as they may be influenced by regression to the mean, improved diagnostic scrutiny, or random variation. Future studies with larger sample sizes and longer observation periods are needed to confirm whether these changes represent true clinical benefits. Compared to the VTE incidence among Chinese hospitalized patients reported by Zhang et al. (3), the post-implementation incidence in this study was lower, likely due to the high standardization of risk assessment and the implementation of preventive measures. The observed clinical outcome improvements also differ significantly from those reported in similar international studies. Amin et al. (19) found that electronic alert systems reduced VTE incidence by approximately 41%, whereas this study demonstrated an even greater reduction. Wang et al. (20) reported that information technology interventions reduced VTE incidence by an average of 50%, a result consistent with the findings of the present study. These differences may stem from three key factors: first, China’s relatively weaker foundation in VTE prevention and control, which provides greater room for improvement through information system interventions; second, the lower baseline VTE incidence in Western hospitals, which limits the potential for further reduction; and third, the integration of multiple innovative functions within this study’s system, particularly the physician-nurse communication module and discharge follow-up module, which are less systematically applied in international research.

### 5.4 System implementation improved healthcare professionals’ VTE knowledge

The results of our study also revealed a significant improvement in healthcare professionals’ knowledge of VTE following system implementation. This included a better understanding of VTE risk assessment scales, increased recognition of the necessity of assessments and physician-nurse collaboration, and an improved appreciation of information technology’s role in VTE prevention. Notably, the proportion of healthcare professionals who perceived VTE prevention and control as an additional workload significantly decreased, suggesting that the system not only improved objective clinical indicators but also positively influenced subjective knowledge and attitudes. The substantial improvement in healthcare professionals’ knowledge in this study may be attributed to the system’s user-friendly interface and real-time decision support, which facilitated a “learning-by-doing” approach. International research highlights different strategies for enhancing healthcare knowledge, often with varied effectiveness. Perret et al. (21) noted that while electronic reminder systems can reinforce knowledge, improvements often lack sustainability without continuous education and feedback mechanisms. In contrast, this study’s system established a self-sustaining cycle of learning while working through real-time decision support and physician-nurse communication modules, which may explain the more pronounced knowledge gains. Additionally, Ashebir et al. (22) emphasized that adherence to clinical guidelines is influenced by knowledge, attitudes, and behaviors. This study’s system addressed all three aspects simultaneously by reducing workload and providing timely feedback, aligning closely with Cabana’s theoretical framework. While international research often employs diversified educational approaches—such as the study published by Zamiri et al. (23), which combined online learning modules, team competitions, and real-time feedback to create a comprehensive learning ecosystem—this study prioritized embedding learning within routine workflows. This embedded approach is particularly suitable for busy clinical environments, where structured training may be challenging to implement effectively.

### 5.5 Comprehensive value of the application of the developed system

The findings of this study indicate that the VTE risk management system, developed within a smart hospital platform, significantly enhanced hospital-wide VTE prevention and control efforts through the integrated application of five functional modules: structured assessment, stratified decision-making, intelligent alerts, physician-nurse communication, and discharge follow-up. Compared to similar domestic and international research, the key innovation of this system lies in its deep integration with the smart hospital platform, enabling seamless physician-nurse collaboration, comprehensive management, and individualized decision support. This integrated information management model aligns with the intelligent VTE management concepts proposed by Zhou et al. (24) and Chiasakul et al. (25). However, this study further extended VTE management to a full-process approach, encompassing admission through discharge. A distinctive feature of this system is its emphasis on physician-nurse communication as a core functional module. This reflects the characteristics of China’s medical system, where physician-nurse collaboration is a critical component of clinical decision-making—a factor that has not been as prominently emphasized in previous related research.

The current system focuses on VTE related risk management and does not integrate the CHA2DS2VASc or HASBLED scores. For patients with comorbid atrial fibrillation, the system prompts physicians to independently perform stroke and bleeding risk assessments and to document the scores in the electronic medical record. Future versions aim to integrate VTE and atrial fibrillation risk modules to achieve more comprehensive thromboembolism risk management.

In clinical practice, patients may receive anticoagulant or antiplatelet drugs for atrial fibrillation while also requiring VTE risk assessment, and these two risk domains often intertwine and cannot be easily separated (26). Our system does not separate them artificially. The bleeding risk assessment table explicitly includes concomitant antiplatelet therapy and concomitant anticoagulant therapy as two of the twenty risk factors. When a patient is on such medications, the system flags these factors and alerts the physician to consider the combined bleeding risk. For VTE prevention, the physician integrates the VTE risk level, the bleeding risk level, and the ongoing antithrombotic regimen. The system provides a reminder that adding pharmacological VTE prophylaxis may increase bleeding risk. In cases where bleeding risk is high, mechanical prophylaxis is preferred, consistent with guideline recommendations (14). This workflow ensures that VTE and atrial fibrillation management are jointly considered without duplication or confusion.

### 5.6 Implications for practice

This system reduces physician workload by delegating initial VTE assessment to nurses, requires only basic IT infrastructure, and benefits from monthly quality meetings and departmental champions. User-friendly design and real-time feedback are key for uptake. When a patient already receiving pharmacological or mechanical prophylaxis is scheduled for surgery, the system triggers an alert at the preoperative assessment point, prompting physicians to reassess both VTE and bleeding risks. Based on the type of surgery, anesthetic method, and half life of the anticoagulant, the system suggests a discontinuation plan, such as stopping low molecular weight heparin 24 h before surgery, and timing for postoperative resumption. For patients at very high bleeding risk, the system may recommend temporary conversion to mechanical prophylaxis alone, followed by reassessment for resumption of pharmacological prophylaxis once the condition of the patient stabilizes postoperatively.

### 5.7 Strengths and limitations

Key strengths of this study include the large sample size, the five-module integrated design, and the combined assessment of both VTE and bleeding risks. Limitations are as follows: (1) the single-center design limits generalizability; (2) the pre-post design without a concurrent control group precludes causal inference and cannot exclude secular trends, Hawthorne effects, or concurrent quality improvement initiatives; (3) the bundled nature of the intervention, with five modules introduced together, prevents us from isolating the effect of any single component; (4) no post-discharge follow-up data are available on long-term VTE and bleeding outcomes; (5) the healthcare professional knowledge survey used a non-validated, self-designed questionnaire. Future multicenter, cluster-randomized controlled trials with extended follow-up are needed to establish definitive effectiveness.

## 6 Conclusion

This smart hospital platform-based VTE risk management system represents a promising and feasible information-driven solution for promoting standardized VTE prevention. Its implementation was associated with substantial gains in process adherence and staff knowledge, alongside favorable trends in clinical outcomes: VTE incidence reduced from 0.316‰ to 0.098‰, bleeding incidence from 0.432‰ to 0.180‰, and length of stay shortened by 1.4 days. Nevertheless, given the uncontrolled study design, these findings should be interpreted as hypothesis-generating rather than confirmatory. The five integrated modules, namely structured assessment, stratified decision-making, intelligent alerts, physician-nurse communication, and discharge follow-up, effectively addressed common barriers to VTE prevention. Future multicenter, cluster-randomized trials with long-term outcome tracking are warranted to establish the definitive effectiveness of this integrated approach.

## Data Availability

The datasets presented in this study can be found in online repositories. The names of the repository/repositories and accession number(s) can be found in the article/supplementary material.
